# Supplementary material for: A high-throughput, flow cytometry approach to measure phase behavior and exchange in biomolecular condensates
Source: Nat Commun. 2026 Jan 15;17:1337. doi: 10.1038/s41467-025-68093-6 (PMC12873376; doi:10.1038/s41467-025-68093-6)
Supplement: Supplementary file 1 — Supplementary Information [file 41467_2025_68093_MOESM1_ESM.pdf]

Supplementary Information

A high-throughput, flow cytometry approach to measure phase behavior and exchange in biomolecular condensates

Yuchen He,[1,#] George M. Ongwae,[1,#] Anupam Mondal,[2] Joel A. Moses,[1] Jeetain Mittal,[2,3,4] and Marcos M. Pires[1,5,*]

[1]Department of Chemistry, University of Virginia, Charlottesville, VA 22904, USA

[2]Artie McFerrin Department of Chemical Engineering, Texas A&M University, College Station, TX 77843, USA

[3]Department of Chemistry, Texas A&M University, College Station, TX 77843, USA

[4]Interdisciplinary Graduate Program in Genetics and Genomics, Texas A&M University, College Station, TX 77843, USA

[5]Department of Microbiology, Immunology, and Cancer, University of Virginia, Charlottesville, VA 22904, USA

[#]These authors contributed equally

*Corresponding author, E-mail: mpires@virginia.edu

Table of Contents:

Supplementary Figure 1 .... **S-3**

Supplementary Figure 2 .... **S-4**

Supplementary Figure 3 .... **S-6**

Supplementary Figure 4 .... **S-7**

Supplementary Figure 5 .... **S-9**

Supplementary Figure 6 .... **S-10**

Supplementary Figure 7 .... **S-12**

Supplementary Figure 8 .... **S-13**

4Supplementary Figure 9 .... **S-14**

Supplementary Figure 10 .... **S-15**

Supplementary Figure 11 .... **S-16**

Supplementary Figure 12 .... **S-17**

Supplementary Figure 13 .... **S-18**

Supplementary Figure 14 .... **S-19**

Supplementary Figure 15 .... **S-20**

Supplementary Figure 16 .... **S-21**

Supplementary Table 1 .... **S-22**

References **S-24**

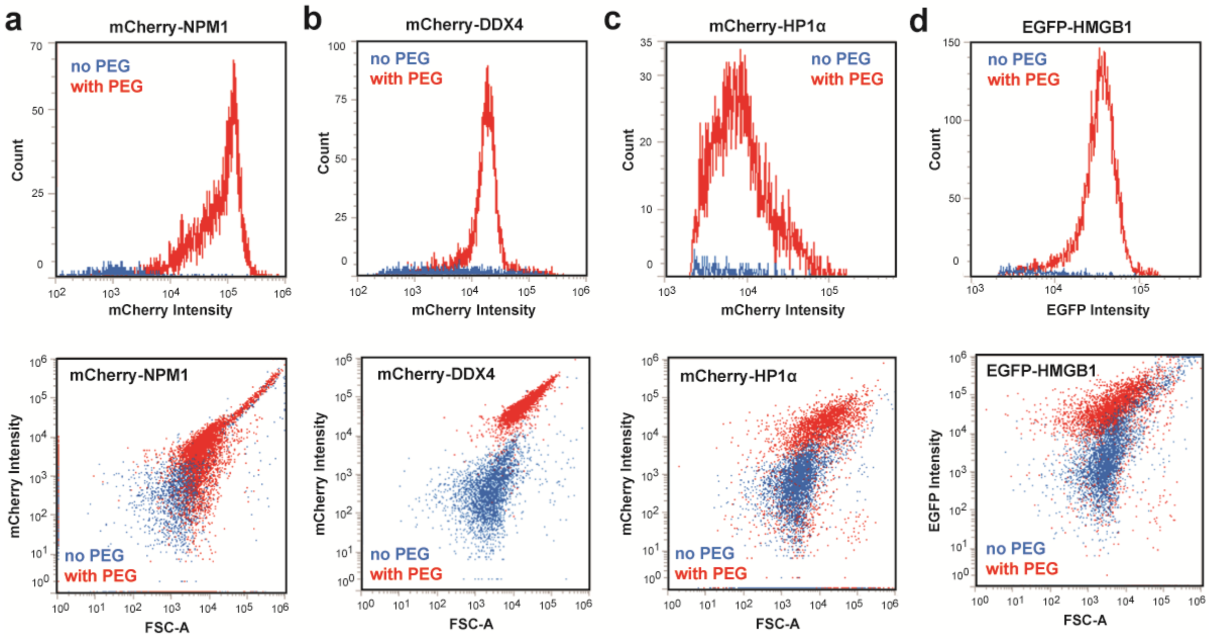

Supplementary Figure 1. Flow cytometry analysis of condensate formation across four scaffold proteins. Top panels: Histograms of fluorescence intensity for **a)** mCherry-NPM1, **b)** mCherry-DDX4, **c)** mCherry-HP1α, and **d)** EGFP-HMGB1. Blue curves represent proteins alone, while red curves represent proteins incubated with PEG8000. In all cases, PEG treatment induces a clear shift toward higher fluorescence intensity, consistent with condensate formation and enrichment of fluorescently tagged proteins per event. Bottom panels: Scatter plots of fluorescence intensity versus FSC-A for the same four proteins under no PEG (blue) and +PEG (red) conditions. PEG treatment results in a clear population shift toward higher FSC-A and fluorescence, indicating increased condensate size and protein content.

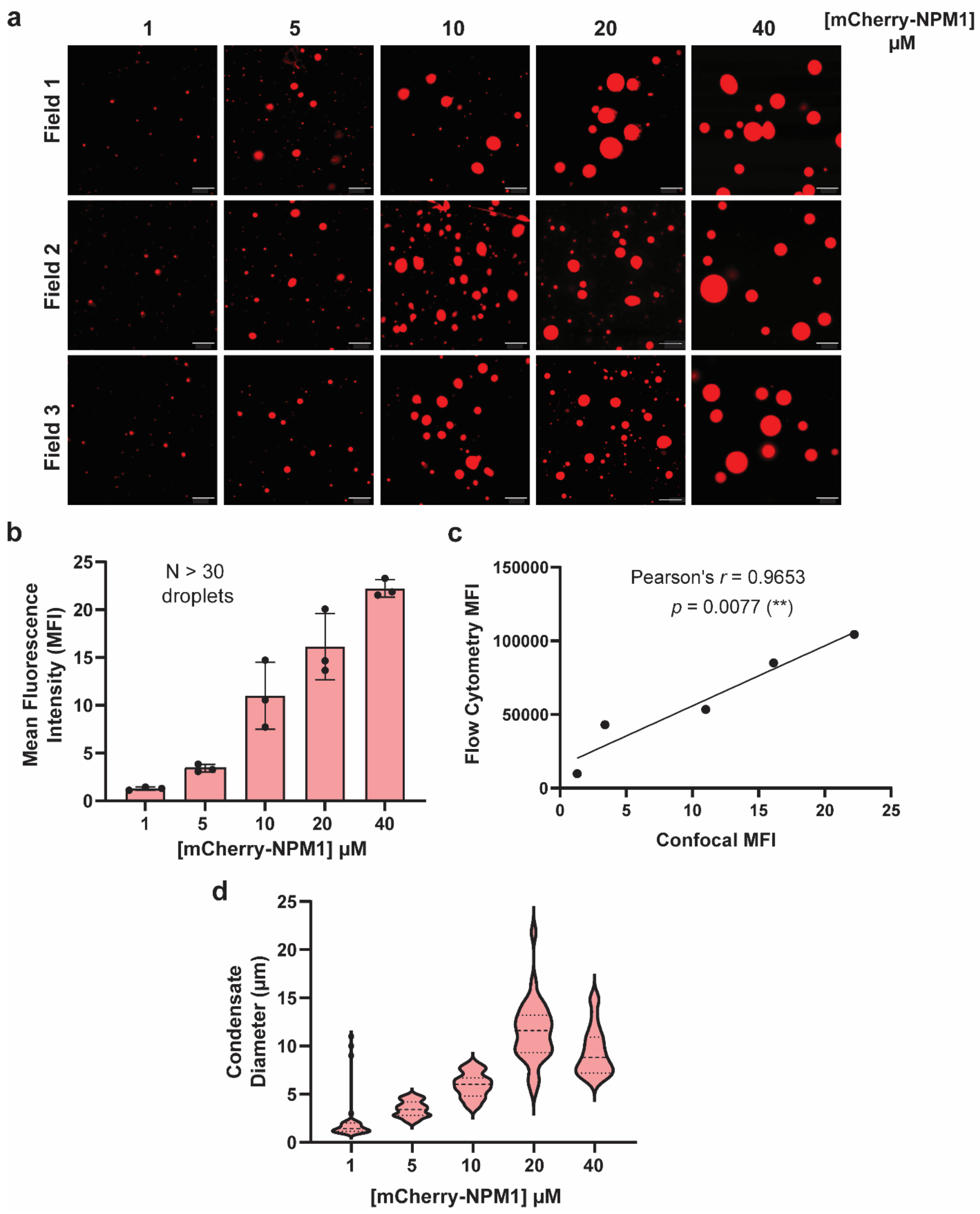

Supplementary Figure 2. Quantitative comparison of confocal imaging and flow cytometry analysis of mCherry-NPM1 condensates. a) Representative confocal

fluorescence images of mCherry-NPM1 condensates at 1, 5, 10, 20, and 40 μM protein concentration. For each concentration, three independent fields of view are shown. Scale bar = 5 μm. **b)** Quantification of mean fluorescence intensity (MFI) per condensate droplet from confocal images using ImageJ segmentation. Data was pooled from three independent fields of view per concentration ($n > 30$ condensates in total; >10 condensates per field). **c)** Correlation between confocal-derived image medians (panel **b**) and median fluorescence measured by flow cytometry on the same samples (**Figure 3d**). Pearson's $r = 0.9653$, $p = 0.0077$ (**), indicating a strong and statistically significant positive correlation between the two methods. **d)** Quantification of condensate diameters from confocal images at the same protein concentrations as in panels **a–b**. Violin plots illustrate the distribution of droplet sizes ($n > 30$ condensates per concentration), showing a general increase in condensate diameter with increasing protein concentration.

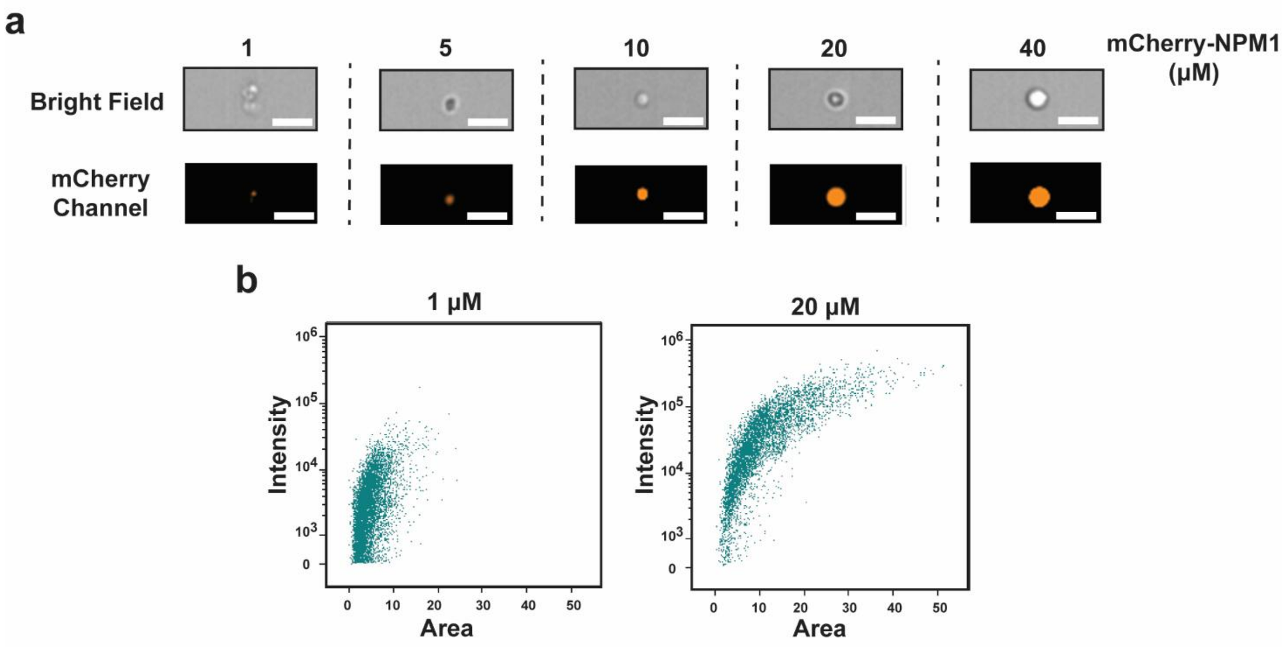

Supplementary Figure 3. Imaging flow cytometry (IFC) analysis of mCherry-NPM1 condensates. a) Representative bright-field and mCherry fluorescence images of single condensates at increasing protein concentrations (1–40 μM). Condensate size visibly increases with higher protein levels. **b)** Scatter plot of surface area (bright field) versus fluorescence intensity (mCherry) for condensates at 1 μM and 20 μM, showing a shift toward larger and brighter particles at higher concentration. This panel illustrates the population-level trends underlying the quantitative measurements presented in **Fig. 4a**. Scale bars = 7 μm.

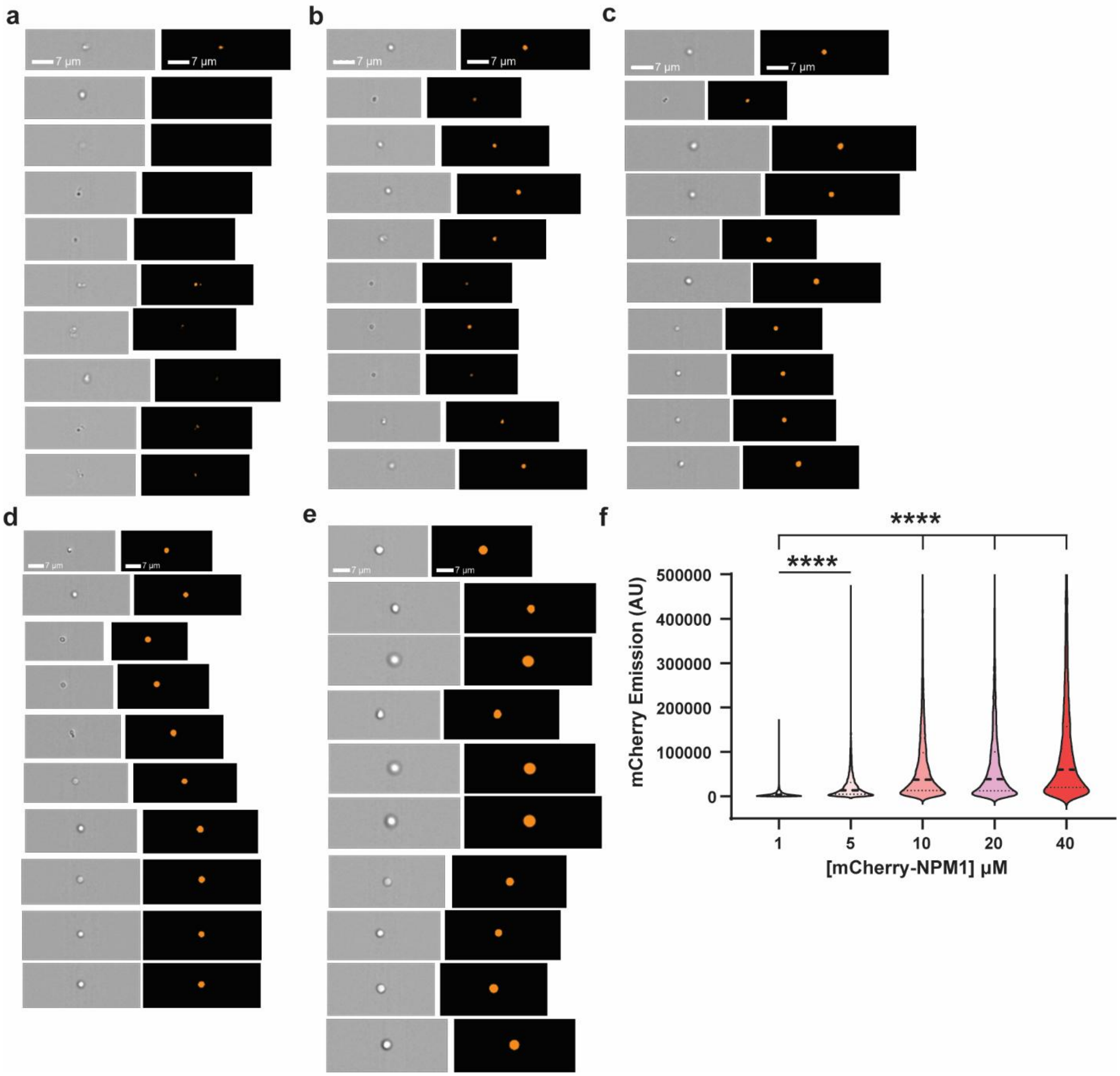

Supplementary Figure 4. Additional representative images and quantification of mCherry-NPM1 condensates captured by imaging flow cytometry (IFC). a-e)

Representative IFC images of mCherry-NPM1 condensates formed at increasing concentrations (1, 5, 10, 20, and 40 μM) in the presence of 10% PEG. Each panel includes images from both the bright field and mCherry fluorescence channels. A general increase in condensate size is observed with higher protein concentrations. Scale bar = 7 μm. **f)** Violin plot showing the distribution of mCherry fluorescence intensity for over 10,000 condensates at each protein concentration. The fluorescence intensity increases with protein concentration, consistent with increased condensate formation. Statistical analysis was performed using unpaired, two-tailed student's *t*-tests. Significance is denoted as follows: $p < 0.0001$ (****). Exact p-values are provided in the Source Data file.

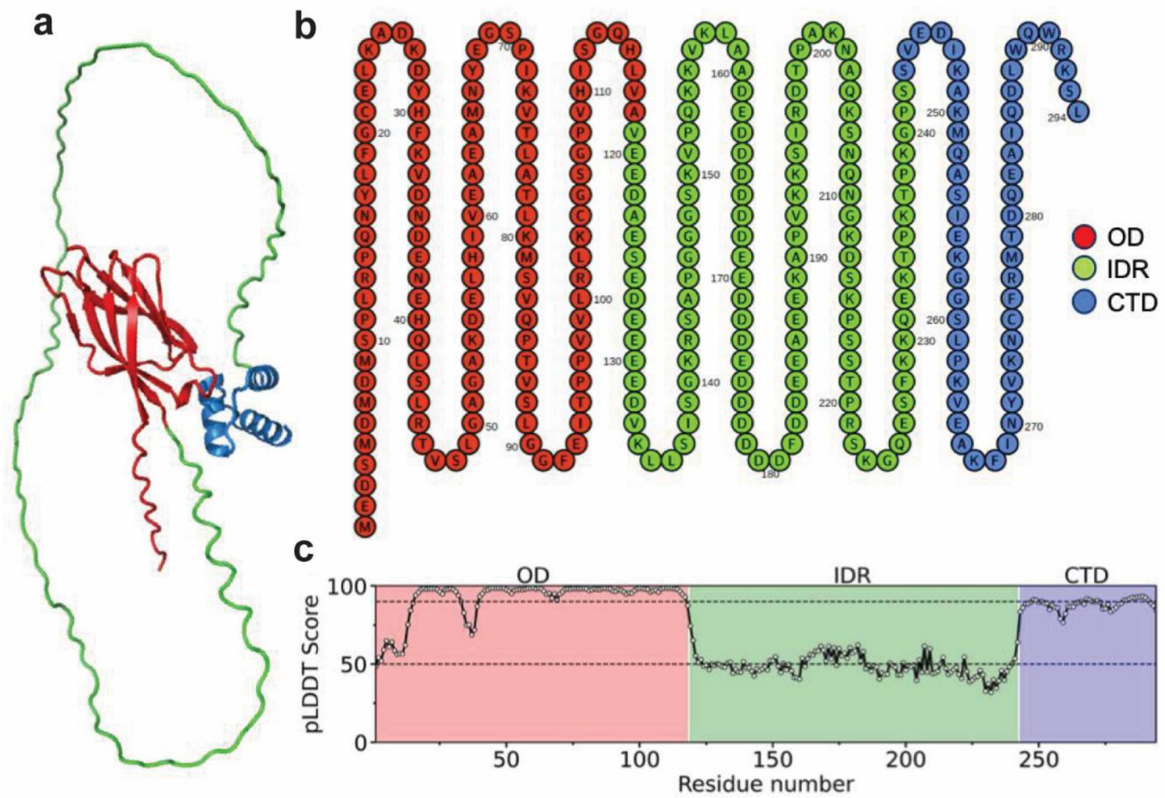

Supplementary Figure 5. Structure and sequence of NPM1 protein used in coarse-grained (CG) simulation. a) Cartoon representation of the Alpha-Fold generated structure NPM1 protein. The N-terminal oligomerization domain (OD) and the C-terminal domain (CTD) of NPM1 are shown by red and blue colors respectively, while the central intrinsically disordered region (IDR) is shown by green color. **b)** Sequence of the full-length NPM1 protein. **c)** pLDDT (predicted local distance difference test) scores for each residue of the AlphaFold model of NPM1 protein. High pLDDT score (above 90, indicated by black dotted line) indicates high confidence in the prediction, in which both the backbone and side chains are typically predicted with high accuracy, where low pLDDT score (below 50, indicated by black dotted line) indicates a region that is naturally highly flexible or intrinsically disordered.

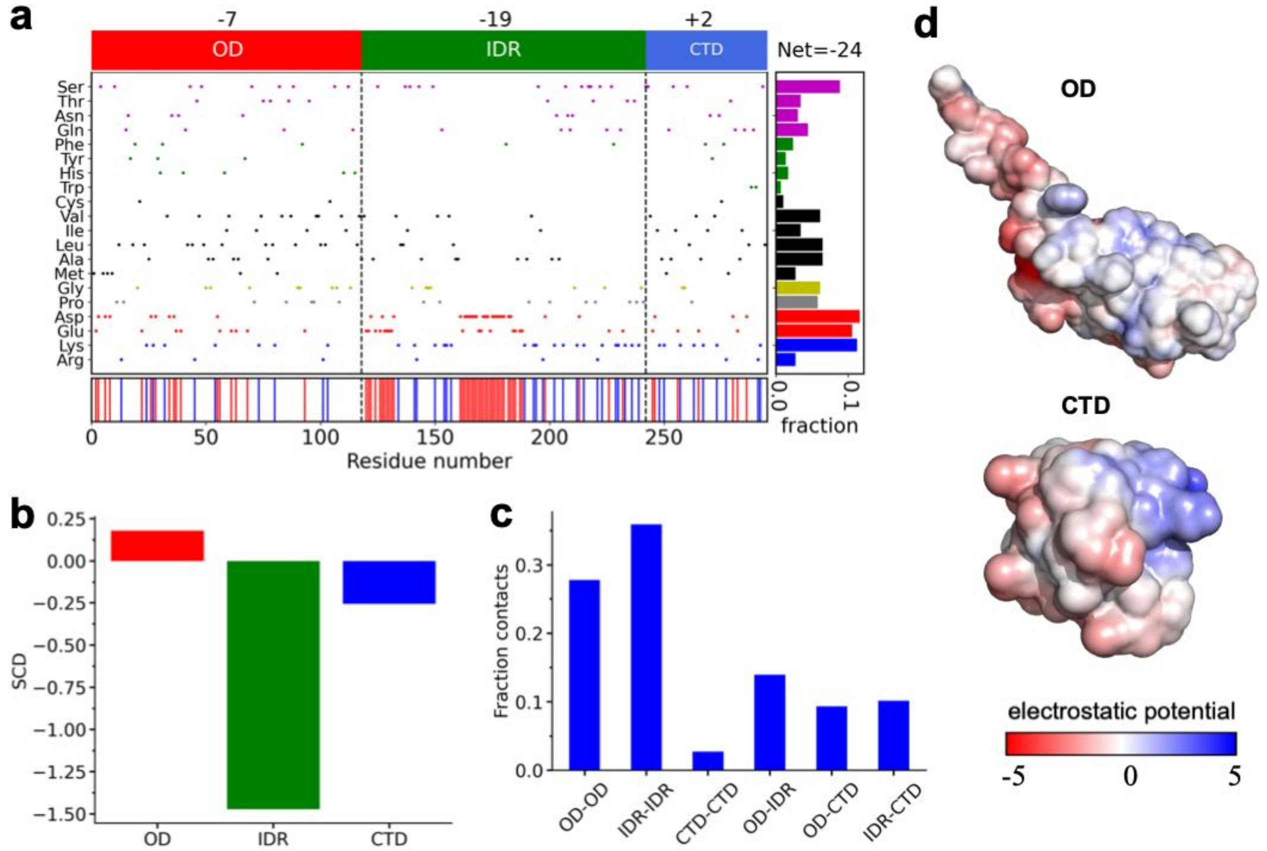

Supplementary Figure 6. Sequence and domain-level charge organization and contact propensity in NPM1 condensates. a) Domain architecture of the full length NPM1 protein, showing the net charge of each domain and distribution of amino acids. Red and blue vertical lines indicate the location of negatively and positively charged residues. **b)** SCD (sequence charge decoration) values for the OD, IDR and CTD regions of NPM1 protein, highlighting the degree of charge segregation.[1] A large negative value of SCD indicates a block-like charge arrangement (which is reflected in the IDR domain in the above panel), while a value close to 0 reflects an alternating charge pattern along the sequence. **c)** Fractional contributions of domain-domain intermolecular contacts (OD–OD, IDR–IDR, CTD–CTD, OD–IDR, OD–CTD, and IDR–CTD) obtained from coarse-grained co-existence simulations of NPM1 condensates. **d)** Electrostatic surface

potentials of the OD and CTD domains of NPM1, represented on the solvent-accessible surface (red=negative, blue=positive, white=neutral). The OD shows prominent positively charged patches, while the CTD exhibits negatively charged regions.

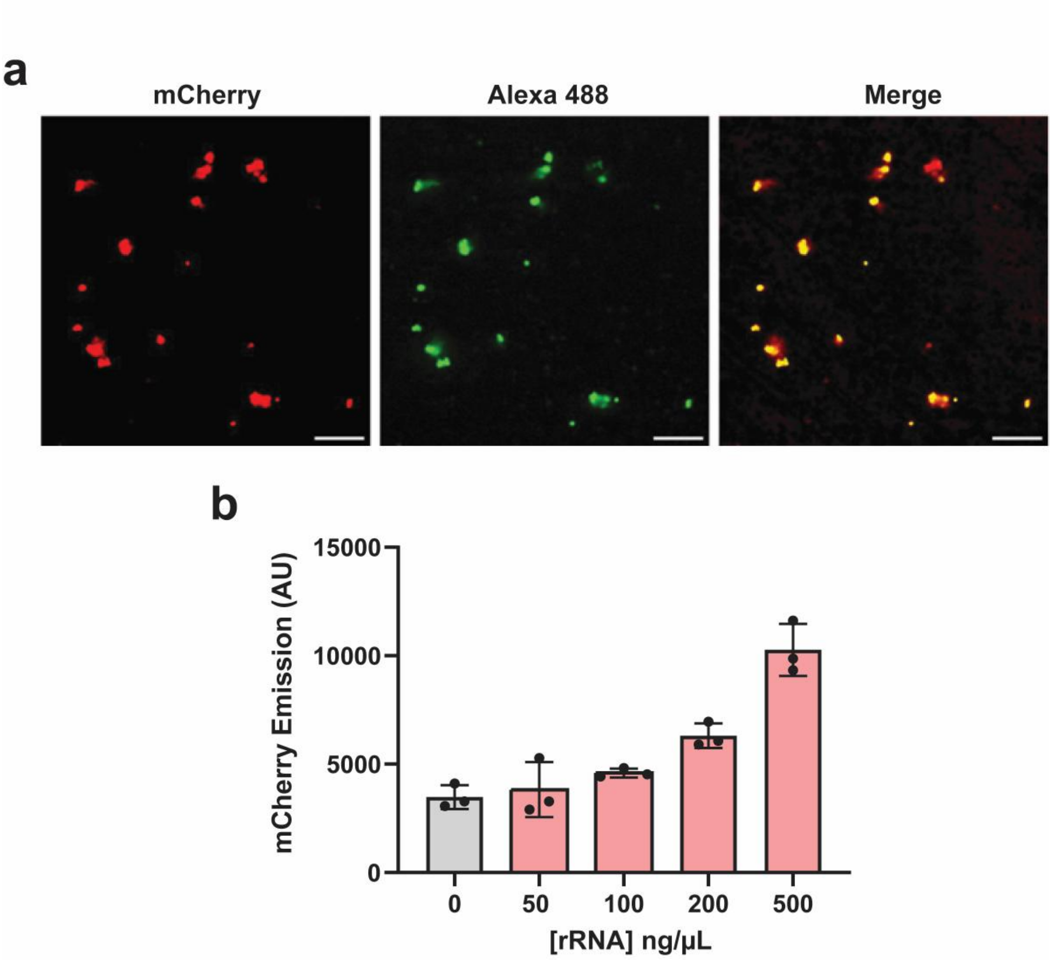

Supplementary Figure 7. RNAs alone can induce condensate formation. **a)** Confocal images of mCherry-NPM1 (20 μM) incubated with RNA (50 ng/μL) in the absence of PEG. In this condition, mCherry-NPM1 forms visible condensate-like assemblies, though their morphology appears irregular and less spherical compared to condensates formed under crowding conditions. Scale bars = 5 μm. **b)** Flow cytometry analysis of mCherry-NPM1 (20 μM) incubated with increasing concentrations of RNA (0–500 ng/μL) in the absence of PEG. Mean fluorescence intensity increased with higher RNA concentrations, indicating a dose-dependent promotion of condensate formation.

a b c d

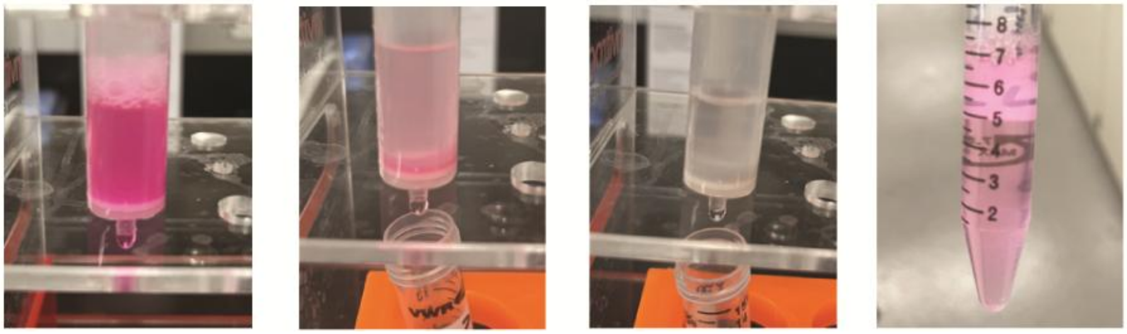

Supplementary Figure 8. Photographic documentation of the purification process of TAMRA-labeled NPM1-Halo proteins. a) During the binding step, NPM1-Halo proteins covalently labeled with TAMRA-chloroalkane (TAMRA-Cl) exhibit a distinct red color on the Ni-NTA resin, indicating successful dye conjugation. **b)** As the wash step proceeds under gravity flow, the red color fades, reflecting the removal of excess unbound dye. **c)** Upon elution with buffer containing high imidazole, most labeled proteins are released from the resin, and the resin becomes nearly colorless. d) The final eluate containing TAMRA-labeled NPM1-Halo proteins appears visibly red in the collection tube, confirming successful labeling and purification. NPM1-Halo proteins labeled with R110-chlooalkane or Coumarin-chloroalkane were purified using the same protocol.

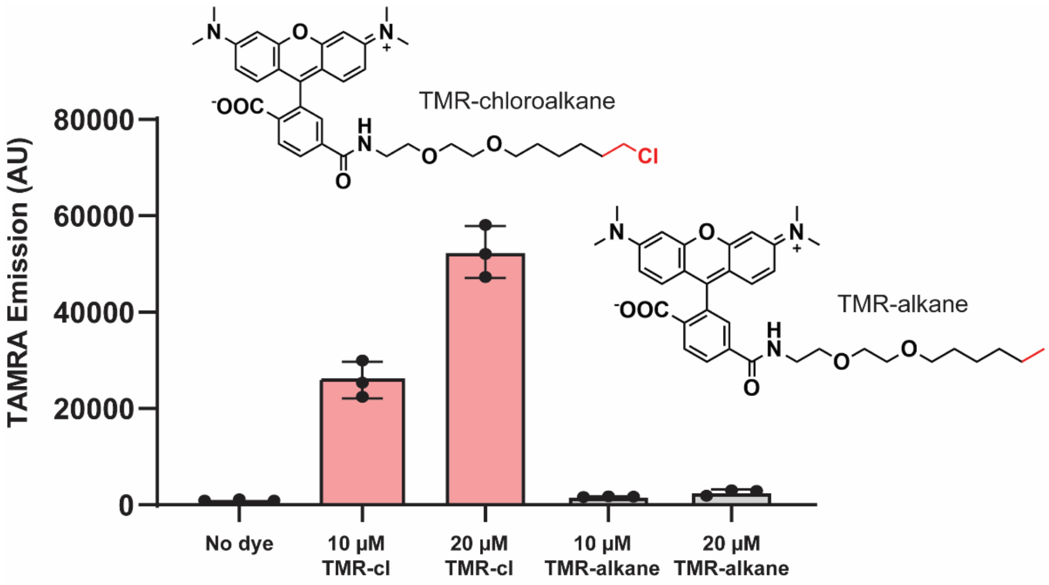

Supplementary Figure 9. TAMRA-chloroalkane but not TAMRA-alkane accumulates in NPM1-Halo condensates. NPM1-Halo protein (20 μM) was incubated with 10% PEG8000 for 30 min to form condensates, then treated with either TAMRA-chloroalkane (TMR-cl) or TAMRA-alkane (TMR-alkane) for 30 min. Flow cytometry showed strong, concentration-dependent fluorescence with TMR-cl, but minimal signal with TMR-alkane, indicating selective accumulation through specific HaloTag binding.

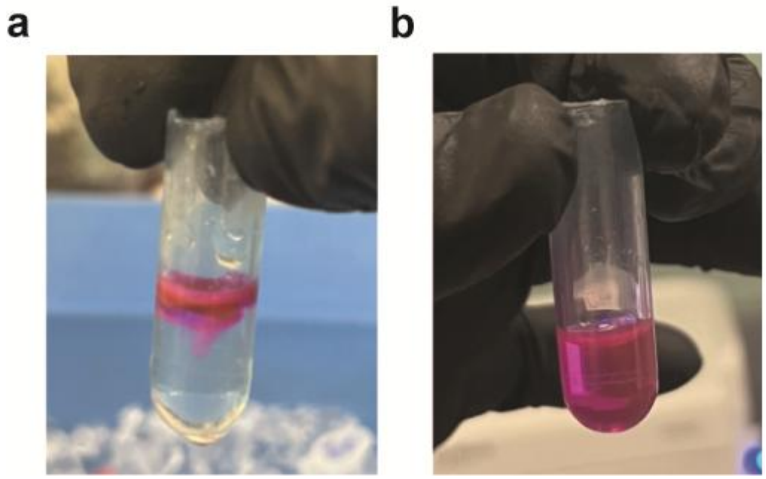

Supplementary Figure 10. Assessment of TAMRA dye mixing in PEG-containing solutions. a) TAMRA-chloroalkane (TAMRA-Cl) dye was added to a 10% PEG solution that had been incubated at room temperature for 3 hours. Without agitation, the dye remained largely undissolved and floated on top of the solution even after 10 minutes, indicating poor spontaneous mixing. **b)** After a brief 2-second vortexing at maximum speed, the dye became fully dispersed, showing homogeneous mixing throughout the PEG solution. These observations confirm that in our condensate dynamic exchange assays, the signal detected by flow cytometry reflects genuine molecular exchange between pre-formed condensates and labeled proteins, rather than incomplete mixing of proteins or dyes in solutions.

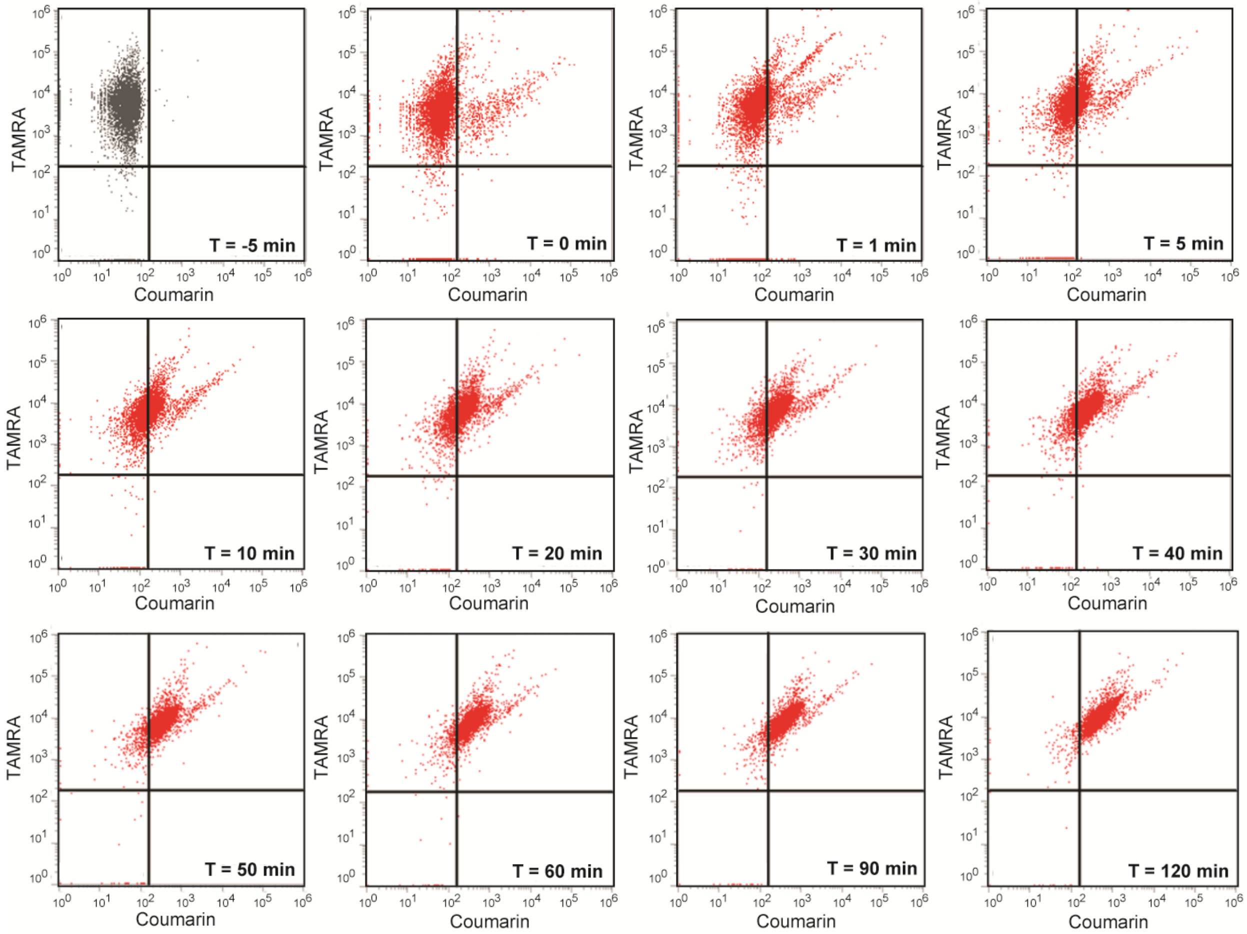

Supplementary Figure 11. Flow cytometry scatter plots show dynamic exchange between pre-formed NPM1-Halo-TAMRA condensates and incoming NPM1-Halo-Coumarin protein. 10 μM pre-formed NPM1-Halo-TAMRA condensates (3 hr aged) were mixed with 10 μM soluble NPM1-Halo-Courmain protein (no PEG) at time 0 min. At T = -5 min, events appear mostly in the TAMRA-positive quadrant, indicating homogeneous red condensates. Upon mixing, populations gradually shift toward the double-positive quadrant (TAMRA+/Coumarin+), reflecting protein exchange over time. By 120 min, over 95% of events reside in the double-positive quadrant, demonstrating efficient exchange which can be captured by flow cytometry.

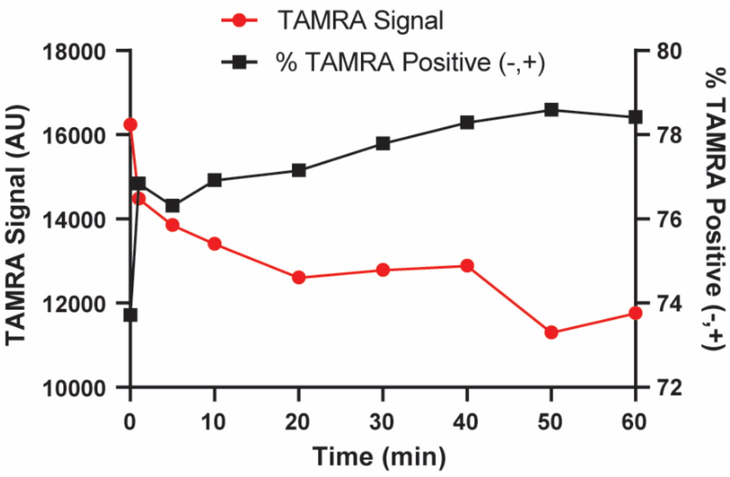

Supplementary Figure 12. Flow cytometry detects dynamic exchange between pre-formed NPM1-Halo-TAMRA condensates and soluble NPM1-Halo-TAMRA protein. 10 µM pre-formed NPM1-Halo-TAMARA condensates (3 hr aged) were mixed with 10 µM soluble NPM1-Halo-TAMRA protein and analyzed over time. Due to identical fluorophores, only the TAMRA-positive (-,+) quadrant was tracked. A slight increase in event count suggests incorporation of soluble protein into condensates. The modest drop in mean TAMRA fluorescence may result from fluorophore dilution, photobleaching, or environmental quenching upon exchange.

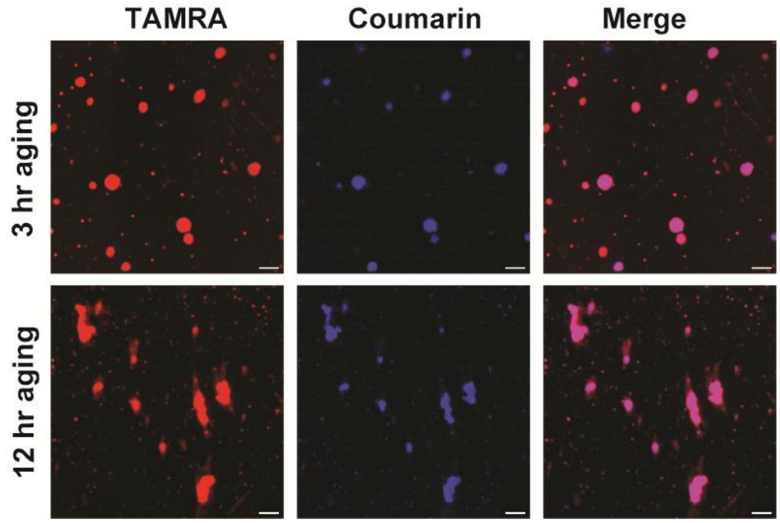

Supplementary Figure 13. Morphological differences in NPM1-Halo condensates following dynamic exchange assays at different aging times. Confocal images show the structural integrity of NPM1-Halo-TAMRA condensates before and after dynamic exchange with NPM1-Halo-Coumarin. **Top row**: 3-hour aged NPM1-Halo-TAMRA condensates maintain a generally round morphology and structural integrity following exchange with NPM1-Halo-Coumarin, indicating a more dynamic and fluid-like state. **Bottom row**: 12-hour aged NPM1-Halo-TAMRA condensates show disrupted morphology and the presence of aggregate-like structures after the same exchange treatment, suggesting increased rigidity or solid-like properties with longer aging. Scale bars = 5 μm.

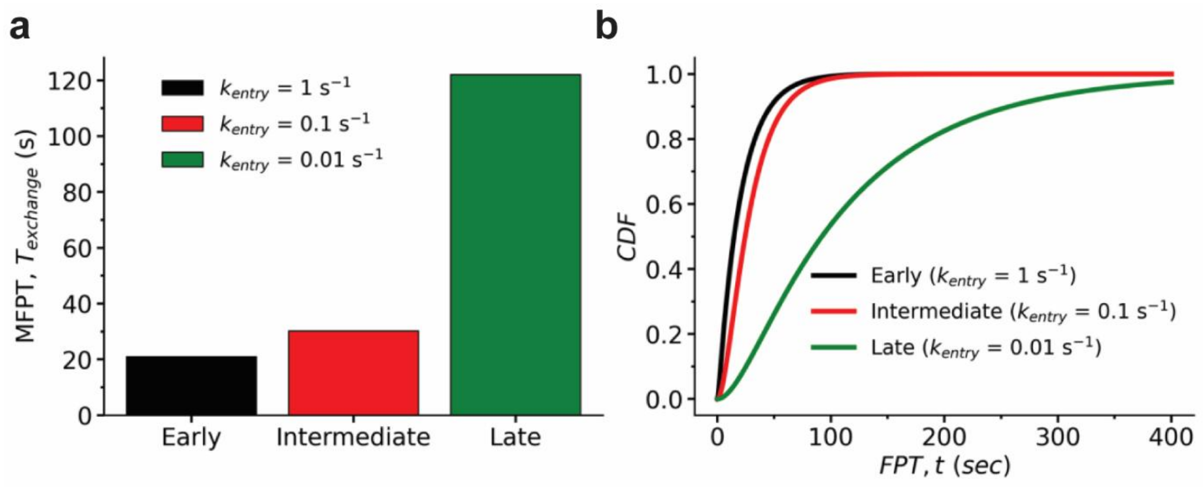

Supplementary Figure 14. Impact of droplet aging via k_{entry} on protein exchange dynamics. a) Mean first-passage time (MFPT) of exchange for early (k_{entry}=1 s^{-1}), intermediate (k_{entry}=0.1 s^{-1}) and late or aged (k_{entry}=0.01 s^{-1}) condensates. **b)** Theoretical cumulative distribution functions (CDFs) of exchange times computed under different aging conditions by varying k_{entry}. Other parameters used for the calculations are: k_{on}=0.05 s^{-1} and k_{bounce}=0.001 s^{-1}.

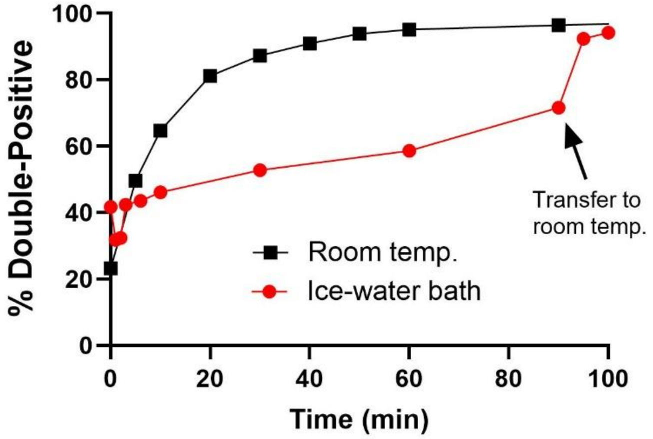

Supplementary Figure 15. Temperature-dependent exchange dynamics within aged NPM1 condensates. Pre-formed NPM1-Halo-TAMRA condensates (10 μM, 3 hr aged) were mixed with 10 μM soluble NPM1-Halo-Coumarin protein and monitored over time using flow cytometry. Samples kept on ice showed a slow increase in double-positive droplets (%), as indicated by the red curve. After 90 minutes, returning the sample to room temperature led to a rapid increase in the double-positive population. For comparison, the black curve represents the same exchange assay performed for a 3 hr aged sample at room temperature (data from Figure 6e). Both conditions eventually reached >90% double-positive droplets, indicating temperature-dependent modulation of exchange dynamics and eventual equilibration.

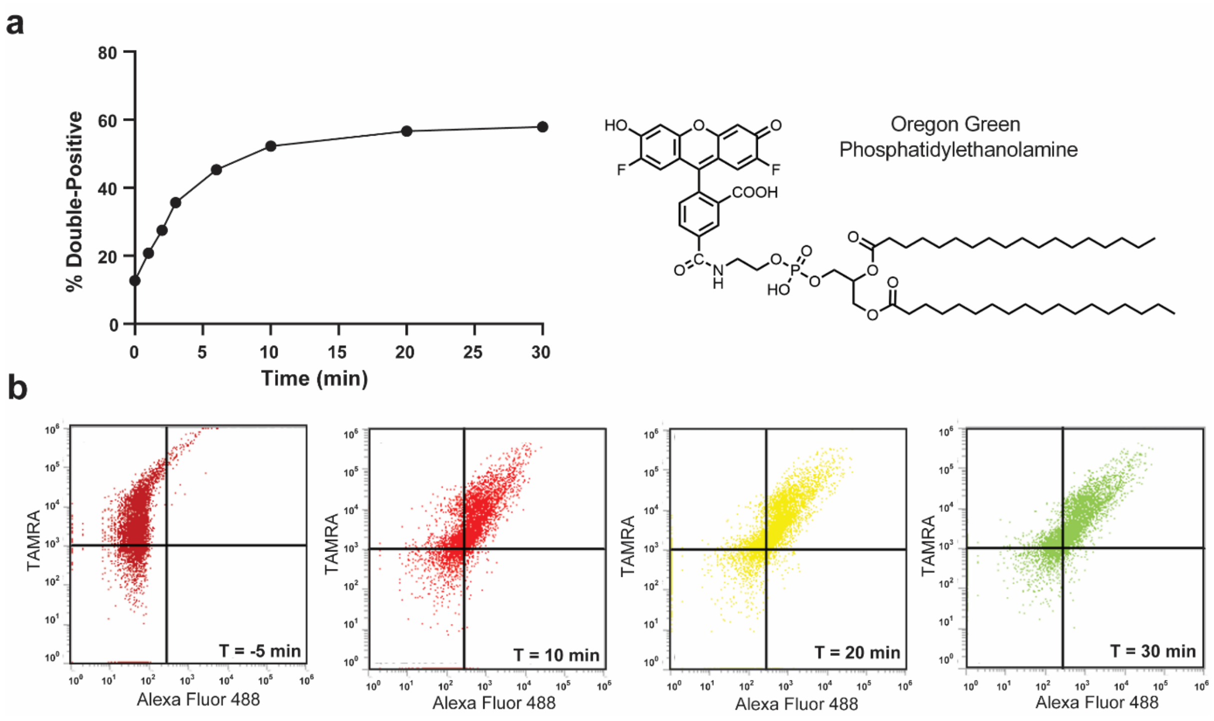

Supplementary Figure 16. Flow cytometry enables real-time tracking of lipid molecule incorporation into pre-formed condensates. a) Quantification of double-positive populations over time during dynamic exchange between pre-formed NPM1-Halo-TAMRA condensates and Oregon Green-conjugated phosphatidylethanolamine (OG-PE). b) Representative scatter plots showing the dynamic progression of molecular exchange. A clear migration of condensate populations into the $TAMRA^{+}$/Alexa Fluor 488^{+} quadrant is observed over 30 minutes after OG-PG addition.

Supplementary Table 1. Corresponding protein sequences

1) pET-28a-HaloTag-NPM1

>Amino acid sequence

MHHHHHHGIEENLYFQSGSGMAEIGTGFPFDPHYVEVLGERMHYVDVGPRDGTPVLF

LHGNPTSSYVWRNIIPHVAPTHRCIAPDLIGMGKSDKPDLGYFFDDHVRFMDAFIEALG

LEEVVLVIHDWGSALGFHWAKRNPERVKGIAFMEFIRPIPTWDEWPEFARETFQAFRTT

DVGRKLIIDQNVFIEGTLPMGVVRPLTEVEMDHYREPFLNPVDREPLWRFPNELPIAGE

PANIVALVEEYMDWLHQSPVPKLLFWGTPGVLIPPAEAARLAKSLPNCKAVDIGPGLNLL

QEDNPDLIGSEIARWLSTLEISGGSGMYTDMEDSMDMDMSPLRPQNYLFGCELKADK

DYHFKVDNDENEHQLSLRTVSLGAGAKDELHIVEAEAMNYEGSPIKVTLATLKMSVQP

TVSLGGFEITPPVVLRLKCGSGPVHISGQHLVAVEEDAESEDEDEEDVKLLGMSGKRS

APGGGNKVPQKKVKLDEDDEDDDEDDEDDEDDDDDDFDEEETEEKVPVKKSVRDTP

AKNAQKSNQNGKDLKPSTPRSKGQESFKKQEKTPKTPKGPSSVEDIKAKMQASIEKG

GSLPKVEAKFINYVKNCFRMTDQEAIQDLWQWRKSLV

>DNA sequence

ATGCACCATCACCATCACCATGGGATCGAGGAAAACCTGTACTTCCAATCCGGTTCT

GGAATGGCCGAAATTGGCACCGGCTTTCCGTTTGATCCGCATTATGTTGAAGTCCT

GGGCGAACGCATGCACTATGTGGATGTGGGTCCGCGTGATGGTACCCCGGTCCTG

TTTCTGCATGGCAACCCGACGAGCAGCTATGTTTGGCGCAATATTATCCCGCATGTT

GCACCGACCCACCGTTGCATTGCCCCGGATCTGATCGGCATGGGCAAAAGCGATA

AACCGGATCTGGGCTATTTCTTTGATGATCACGTGCGCTTTATGGATGCGTTTATTGA

AGCCCTGGGCCTGGAAGAAGTGGTTCTGGTTATCCATGATTGGGGCAGCGCACTG

GGTTTTCACTGGGCCAAACGCAACCCGGAACGTGTTAAAGGCATTGCGTTTATGGA
ATTTATTCGCCCGATCCCGACCTGGGATGAATGGCCGGAATTTGCCCGTGAAACGT
TTCAGGCGTTTCGCACCACGGATGTGGGCCGTAAACTGATCATCGATCAGAACGTT
TTCATCGAGGGTACGCTGCCGATGGGCGTCGTGCGTCCGCTGACGGAAGTTGAAA
TGGATCATTATCGTGAACCGTTTCTGAATCCGGTCGATCGCGAACCGCTGTGGCGT
TTTCCGAACGAACTGCCGATTGCGGGCGAACCGGCCAATATCGTCGCGCTGGTTG
AAGAATATATGGATTGGCTGCACCAGAGCCCGGTCCCGAAACTGCTGTTTTGGGGT
ACCCCGGGCGTGCTGATTCCGCCGGCCGAAGCGGCCCGCCTGGCGAAAAGCCTG
CCGAATTGTAAAGCCGTGGATATCGGCCCGGGCCTGAACCTGCTGCAGGAAGATAA
TCCGGATCTGATTGGCAGCGAAATCGCGCGTTGGCTGAGCACGCTGGAAATCAGC
GGCGGTTCTGGAATGTACACGGATATGGAAGACTCGATGGATATGGACATGAGTCC
TCTTAGGCCTCAGAACTACCTTTTCGGCTGTGAACTAAAGGCTGACAAAGACTATCA
CTTTAAAGTGGATAATGATGAAAATGAGCACCAGTTGTCATTAAGAACGGTCAGTTTA
GGAGCAGGGGCAAAAGATGAGTTACACATCGTAGAGGCAGAAGCAATGAACTATGA
AGGCAGTCCAATTAAAGTAACACTGGCAACTTTGAAAATGTCTGTACAACCAACAGT
TTCCCTAGGGGGCTTTGAAATTACACCACCTGTGGTCTTACGGTTGAAGTGTGGTT
CAGGGCCTGTGCACATTAGTGGACAGCATCTAGTAGCTGTAGAGGAAGATGCAGAG
TCTGAAGATGAAGATGAGGAGGACGTAAAACTCTTAGGCATGTCTGGAAAGCGATC
TGCTCCTGGAGGTGGTAACAAGGTTCCACAGAAAAAAGTAAAACTTGATGAAGATG
ATGAGGACGATGATGAGGACGATGAGGATGATGAGGATGATGATGATGATGATTTTG
ATGAAGAGGAAACTGAAGAAAAGGTCCCAGTGAAGAAATCTGTACGAGATACCCCA
GCCAAAAATGCACAAAAATCAAACCAAAATGGAAAAGACTTAAAACCATCAACACCG
AGATCAAAGGGTCAAGAGTCCTTCAAAAAACAGGAAAAGACTCCTAAAACACCAAA

AGGACCTAGTTCTGTAGAAGACATTAAGGCAAAAATGCAAGCAAGTATAGAAAAAGG

CGGTTCTCTTCCCAAAGTGGAAGCCAAGTTCATTAATTATGTGAAGAATTGTTTCCG

GATGACTGACCAGGAGGCTATTCAAGATCTCTGGCAGTGGAGGAAATCTCTTGTTTA

A

References

1. Sawle, L. & Ghosh, K. A theoretical method to compute sequence dependent configurational properties in charged polymers and proteins. *The Journal of chemical physics* **143** (2015).
